# COVID-19 Hierarchical Classification Using a Deep Learning Multi-Modal

**DOI:** 10.3390/s24082641

**Published:** 2024-04-20

**Authors:** Albatoul S. Althenayan, Shada A. AlSalamah, Sherin Aly, Thamer Nouh, Bassam Mahboub, Laila Salameh, Metab Alkubeyyer, Abdulrahman Mirza

**Affiliations:** 1 Information Systems Department, College of Computer and Information Sciences, King Saud University, Riyadh 11543, Saudi Arabia; saalsalamah@ksu.edu.sa (S.A.A.); amirza@ksu.edu.sa (A.M.); 2 Information Systems Department, College of Computer and Information Sciences, Imam Mohammed Bin Saud Islamic University, Riyadh 11432, Saudi Arabia; 3 National Health Information Center, Saudi Health Council, Riyadh 13315, Saudi Arabia; 4 Digital Health and Innovation Department, Science Division, World Health Organization, 1211 Geneva, Switzerland; 5 Institute of Graduate Studies and Research, Alexandria University, Alexandria 21526, Egypt; sherin.aly.au@gmail.com; 6 Trauma and Acute Care Surgery Unit, College of Medicine, King Saud University, Riyadh 12271, Saudi Arabia; tnouh@ksu.edu.sa; 7 Clinical Sciences Department, College of Medicine, University of Sharjah, Sharjah P.O. Box 27272, United Arab Emirates; bhmahboub@dha.gov.ae; 8 Sharjah Institute for Medical Research, University of Sharjah, Sharjah P.O. Box 27272, United Arab Emirates; lisalameh@yahoo.com; 9 Department of Radiology and Medical Imaging, King Khalid University Hospital, King Saud University, Riyadh 12372, Saudi Arabia; dr.metab@gmail.com

**Keywords:** artificial intelligence, COVID-19, CXR, hierarchical, deep learning, multi-modal, diagnosis, image classification, multi-classes, pneumonia

## Abstract

Coronavirus disease 2019 (COVID-19), originating in China, has rapidly spread worldwide. Physicians must examine infected patients and make timely decisions to isolate them. However, completing these processes is difficult due to limited time and availability of expert radiologists, as well as limitations of the reverse-transcription polymerase chain reaction (RT-PCR) method. Deep learning, a sophisticated machine learning technique, leverages radiological imaging modalities for disease diagnosis and image classification tasks. Previous research on COVID-19 classification has encountered several limitations, including binary classification methods, single-feature modalities, small public datasets, and reliance on CT diagnostic processes. Additionally, studies have often utilized a flat structure, disregarding the hierarchical structure of pneumonia classification. This study aims to overcome these limitations by identifying pneumonia caused by COVID-19, distinguishing it from other types of pneumonia and healthy lungs using chest X-ray (CXR) images and related tabular medical data, and demonstrate the value of incorporating tabular medical data in achieving more accurate diagnoses. Resnet-based and VGG-based pre-trained convolutional neural network (CNN) models were employed to extract features, which were then combined using early fusion for the classification of eight distinct classes. We leveraged the hierarchal structure of pneumonia classification within our approach to achieve improved classification outcomes. Since an imbalanced dataset is common in this field, a variety of versions of generative adversarial networks (GANs) were used to generate synthetic data. The proposed approach tested in our private datasets of 4523 patients achieved a macro-avg F1-score of 95.9% and an F1-score of 87.5% for COVID-19 identification using a Resnet-based structure. In conclusion, in this study, we were able to create an accurate deep learning multi-modal to diagnose COVID-19 and differentiate it from other kinds of pneumonia and normal lungs, which will enhance the radiological diagnostic process.

## 1. Introduction

Healthcare systems worldwide are seriously endangered by a type of viral pneumonia known as COVID-19. As the virus that causes COVID-19 has a high pathogenicity similar to SARS-CoV, it is known as severe acute respiratory syndrome coronavirus-2 (SARS-CoV-2) [1]. Respiratory insufficiency can arise from the virus’s impact on the patient’s respiratory system, specifically the lungs. As of 11 February 2024, the World Health Organization (WHO) has received reports of 774,631,444 confirmed cases of COVID-19, as well as 7,031,216 deaths from respiratory failure and injury to other major organs [2]. China reported its first instances of the virus to the WHO in December 2019. Hospitalization rates have increased globally due to the disease’s rapid spread [3]. However, while the peak of the initial crisis may be over, the world is still dealing with the lasting effects of the COVID-19 pandemic. Continuous research in this field is important to improving the diagnosis of future respiratory disease pandemics.

RT-PCR testing is a standard, highly sensitive method for diagnosing COVID-19 [4]. The method has some limitations that make its use a challenge in critical cases [5]. In emergency situations, radiologists can assist physicians in diagnosing lung diseases by using imaging modalities such as chest computed tomography (CT scans) or CXR. RT-PCR limitations can be overcome with accurate analysis for radiological imaging. These procedures are challenging due to time constraints and the large number of patients. Furthermore, erroneous diagnoses can result from radiologists with different levels of expertise [6].

Chest radiography is a significant diagnostic tool [7], particularly considering the accuracy with which CT scans can identify and detect pneumonia; however, in this context, CXR may be more appropriate. This is due to several reasons: CXR is inexpensive, simple, portable, available, and the most widely used tool for diagnosing the lungs [8]; it has also been recommended by the American College of Radiology (ACR) to use CXR imaging as a certified modality to reduce the possibility of disease transmission [9].

**Figure 1:**
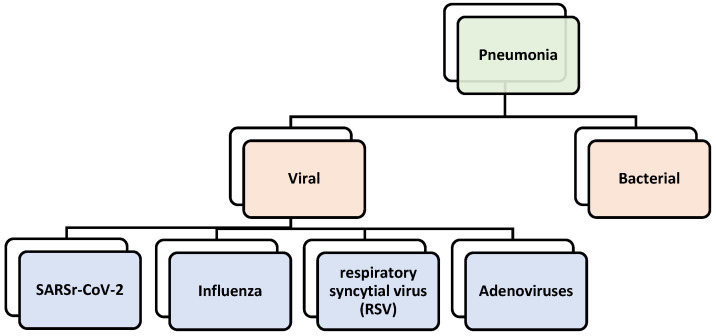
Proposed hierarchal class structure of pneumonia.

It is important to note that all kinds of pneumonia that infect the lungs are classified in a hierarchical structure by the International Statistical Classification of Diseases (ICD). COVID-19, scientifically referred to as SARSr-CoV-2, was added to the hierarchical viral family in the ICD 10th revision (ICD-10) [10]. Figure 1 presents the types of pneumonia for which datasets were obtained that are organized in a hierarchical structure. There are eight classes in total (with a normal class), six of which are leaf nodes.

As illustrated previously, the hierarchical structure of COVID-19 indicates that this is a hierarchical classification problem. The classes in high-level nodes at hierarchical levels are known as coarse-grained nodes because they have unique features that will be transmitted to their child nodes along with all the features from their parent node. Furthermore, the final level of nodes in the structure, the leaf node, is referred to as fine-grained since it lacks descendants and inherits all of its parent’s features.

Applying high-accuracy artificial intelligence (AI) models to diagnose medical imaging problems is a current trend in healthcare. Convolutional neural networks are able to detect and learn the significant details that radiologists find difficult to recognize with their naked eyes [11]. It produces promising results for learning complex problems in radiology [12]. Many of the previously reviewed studies [13] have employed deep learning models to diagnose and detect COVID-19 pneumonia utilizing medical imaging in a theoretical manner that cannot be implemented clinically. CT scans have primarily been considered as the main radiological imaging modality for all infected cases during the ongoing pandemic. In addition, most studies on COVID-19 image classification are misleading due to the use of binary classification and larger samples for COVID-19 classification. Despite this, the ability of AI systems to differentiate between various classes is increasing as they learn from a greater number of classes. Most approaches use a flat structure, while pneumonia naturally falls into a hierarchical structure. Despite several techniques used for COVID-19 detection and classification, limited research has addressed multi-modal deep learning models for heterogeneous data types. Most existing models focus on a single feature modality, while multi-modal features combine multiple aspects of COVID-19 health information, contributing to superior disease diagnostic processes. 

In several fields, especially in diagnosis by medical assistants, deep learning approaches have accomplished significant advances in multi-modal structures by learning features from different sources of data [14]. This clearly explains the effectiveness of adding various medical data in addition to CXR images for the diagnostic process.

One variant of the conventional flat classification problem is hierarchical classification (HC). In a flat classification approach, cases are categorized into classes without following any predefined structure. 

Proving that hierarchical classification is more effective than flat classification in this domain is not the purpose of this work, as this has already been addressed in the literature [15]. In this work, we investigated how clinical data affect COVID-19 classification utilizing CXR images with a hierarchical classification framework to detect different types of pneumonia caused by multiple pathogens and differentiated them from normal lungs. To achieve this, we collected a private, imbalanced dataset in which some types of pneumonia are much more common than others. To this end, we applied variants of the GAN model to balance the class distribution. We first applied multi-modal hierarchical classification utilizing a deep learning approach for two predefined models in a hierarchical structure using a hybrid approach to the CXR images; then, the medical tabular data were added using early fusion. It is important to note that the newly released WHO normative guidance for applying artificial intelligence in health recognizes the risks, and we are in compliance with their recommendations for safe and effective implementation [16,17].

This paper is organized as follows: Section 2 covers the related works in the literature. The proposed methodology and details of the dataset used in this paper and its analysis, as well as the techniques used to preprocess either the CXR images or the tabular dataset, are discussed in Section 3. After that, Section 4 details the proposed architecture of hierarchical multi-modals and the training procedure. The experimental setup and obtained results and a discussion are summarized in Section 5. Finally, the conclusions of the current work and some possibilities for future works are described in Section 6.

## 2. Related Work

As a result of the COVID-19 pandemic, COVID-19 medical image classification has recently attracted a lot of scientific interest. Researchers from a variety of disciplines have developed deep learning detection and classification models to diagnose COVID-19 reliably and quickly by analyzing radiological images. We published a review paper called “Detection and Classification of COVID-19 by Radiological Imaging Modalities Using Deep Learning Techniques: A Literature Review” [13], which attempts to explore all related remarkable works in the literature and study and analyze them to explain how most current key approaches to the COVID-19 classification challenge have gaps and untapped potential. In addition, in the aforementioned paper, we provided some recommendations addressing various aspects that may help researchers in this field.

## 3. Proposed Methods and Materials

**Figure 2:**
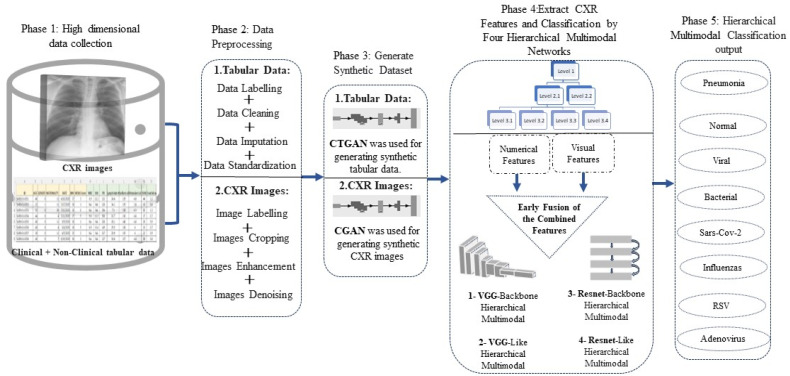
Proposed framework for multi-modal classification of CXR images and tabular data.

The proposed approach that is applied in this study consists of five phases, as demonstrated in Figure 2. These phases are as follows: collecting the required dataset (CXR images and patient medical data in tabular format), preparing and preprocessing the collected dataset, generating a synthetic dataset to balance the data, feeding the preprocessed dataset into a hierarchal multi-modal network, and evaluating the classification output. The details of each phase are described in the following sections.

### 3.1. Dataset Description

Our study used two anonymized private datasets: (I) The first dataset was collected from the database at King Khalid University Hospital, Riyadh, KSA, under Institutional Review Board (IRB) approval (E-21-5939) in collaboration with Dr. Thamer Nouh and Dr. Metab Alkubeyyer. It contains 3326 patients of normal, bacterial, SARS-CoV-2, influenza, respiratory syncytial virus (RSV), and adenovirus cases, as shown in Table 1. (II) The second dataset was obtained from Rashid Hospital, Dubai, UAE, reviewed and approved by the Dubai Scientific Research Ethical Committee (DSREC), Dubai Health Authority (DHA); IRB approval was acquired (DSREC-12/2021_01) in collaboration with Dr. Bassam Mahboub and Dr. Laila Salameh, containing 1218 SARS-CoV-2 patients, as shown in Table 2. Efforts were made to obtain data from different sources to improve the model’s generalization capabilities.

**Figure 3:**
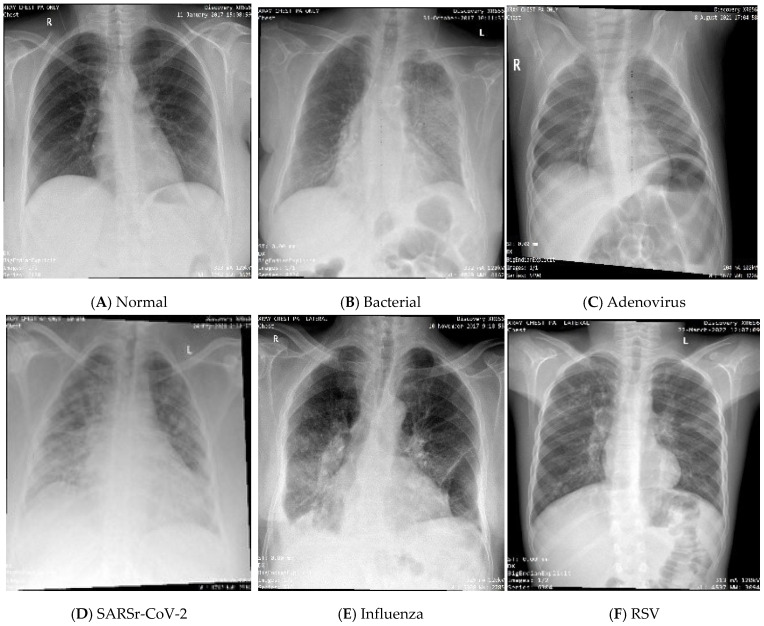
CXR samples of the dataset.

To obtain the datasets from the hospital databases, a physician gathered the required viral patients by searching for the desired test name and range of years. While there is no test for bacterial infection, the patients whose diagnosis contains the word “bacterial pneumonia” were selected as cases for the bacterial class. It is important to note that the data in the normal category were collected from a patient who was scheduled for surgery to ensure that the patient’s lungs were healthy. The CXR images from the first dataset were produced using an Optima XR240amx, General Electric Healthcare from Chicago, United States. All CXR images in datasets (I) and (II) are posterior–anterior (PA), and anterior–posterior (AP) views were included. While there were CXR images of both lateral views, the date gap between the diagnosis and obtaining the CXR images was more than 48 h, and/or those that did not have tabular data were excluded. Some samples of the dataset for different classes are shown in Figure 3. The process of obtaining IRB approval from hospitals and collecting, cleaning, and organizing the data took approximately a full year. The COVID-19 cases were collected for the patients that visited from the year 2019 to the year 2022, while the remaining cases were collected for patients from the year 2014 to the year 2023.

The dataset consists of CXR images with the corresponding medical tabular data for each patient. It includes demographic, vital signs, clinical, and medication data; the attributes in the data record are described in Table 3. The tabular dataset includes 644 features with categorical and numerical data types. Some of the features have been removed since they were not significant in diagnosing pneumonia. In addition, there are a total of 60 different nationalities represented in the patient sample for the 4523 patients who make up the entire dataset. 

The MEWS (modified early warning score) feature is a clinical tool used in healthcare settings to assess a patient’s vital signs. More hospitals are currently using it to help track changes between each set of vitals [18]. The MEWS score typically consists of several physiological parameters including blood pressure, body temperature, pulse rate, respiratory rate, and the AVPU (A = Awake, V = Verbal, P = Pain, U = Unresponsive) score, which is used to determine a patient’s level of consciousness. A score is given for each parameter based on specified standards. The overall MEWS score is then determined by summing the scores for each parameter [19]. According on their MEWS score, patients may be classified into risk categories using the MEWS scoring system: Normal: 0–1 score, Low Risk: 2–3 score, Moderate Risk: 4–6, High Risk: 7–8, Critical: >8. The concern for clinical deterioration increases as the MEWS score rises.

With the assistance of a knowledgeable pharmacist, medication prescriptions were also limited to the most fundamental categories without doses, which helped to decrease the enormous number of medications from 3500 to 614.

### 3.2. Data Analysis

Statistical analysis was conducted with python libraries related to statistical testing. The *t*-test, Kruskal–Wallis test, and chi-squared test, accessed from the “scipy.stats” module, were applied for continuous, categorical, and binary categorical values, respectively. The reported significance levels were two-sided, and the statistical significance level was set to 0.05. The categorical features were expressed as frequency (%), mean (µ), and standard deviation (σ) for continuous features. Table 4 represents a comparison of the features between the two patient groups for the raw dataset, the first with COVID-19 and the second for all remaining classes.

As we can see, there is a significant difference in age, gender, and BMI between the two groups (all *p* < 0.001). The MEWS score shows a significant difference (*p* < 0.001), and an abnormal MEWS score was more often observed in COVID-19 patients. Significant differences were not found in some lab tests between the two groups, including tHb (*p* = 0.729), Potassium Lvl (*p* = 0.606), Alk Phos (*p* = 0.187), Bili Total (*p =* 0.477), INR (*p =* 0.680), LDH (*p* = 0.553), Ferritin Lvl (*p =* 0.419), BNP (*p =* 0.569), and Vitamin D 25 OH (*p* = 0.818). WBC, Plt, Lymph Auto #, Sodium Lvl, BUN, Creatinine, Albumin Lvl, ALT, and Total CK were significantly different between the two groups (*p* < 0.001). In addition, AST, Procalcitonin, CRP, Hgb A1c, and D-Dimer also showed significant differences (*p* = 0.021, *p* = 0.036, *p* = 0.009, *p* = 0.001, and *p* = 0.030, respectively). Although medications were observed in COVID-19 patients, they were not statistically different compared with those in the non-COVID-19 group.

**Figure 4:**
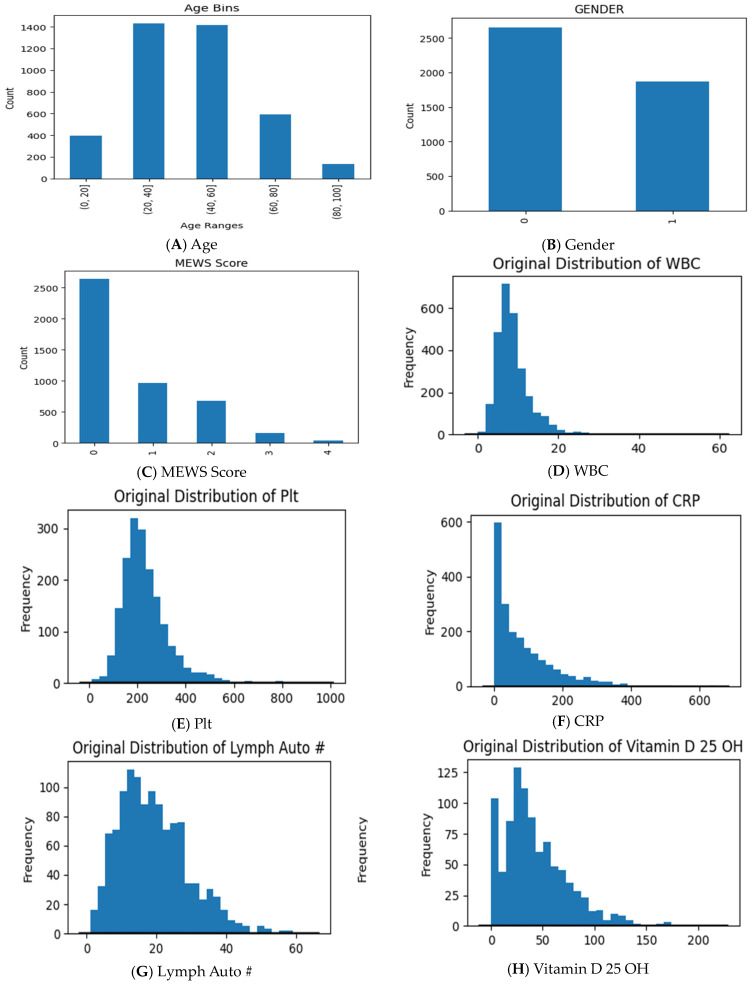
Exploratory analysis of the numeric features.

There are almost 59.5% Saudi citizens among all classes. The distribution of the data was also shown using a number of visualizations. Figure 4 shows the distribution of some continuous and categorical features; many medical features were observed. Chart (A) in Figure 4, showing the age distribution across all datasets, reveals that the majority of patients fall within the 20–60-year age range, followed by those aged between 60 and 80, while the smallest sample size belongs to patients over 80 years old. The distribution of patient gender is presented in chart (B), where we find that the total females (represented by 1) are 1869 and the total males (represented by 0) are 2654 patients. Approximately 57% of the total patients had a normal MEWS score, while 1.2% from all classes were critical cases with an abnormal MEWS score, as shown in chart (C). Charts (D), (E), and (F) show that the majority of patients typically have a normal white blood cell (WBC) count, platelet (PLT) count, and C-reactive protein (CRP) result, respectively. Vitamin D 25 OH in chart (H) shows a right-skewed distribution, and the lymphocyte percentage (Lymph Auto #) in chart (G) shows an almost normal distribution.

**Figure 5:**
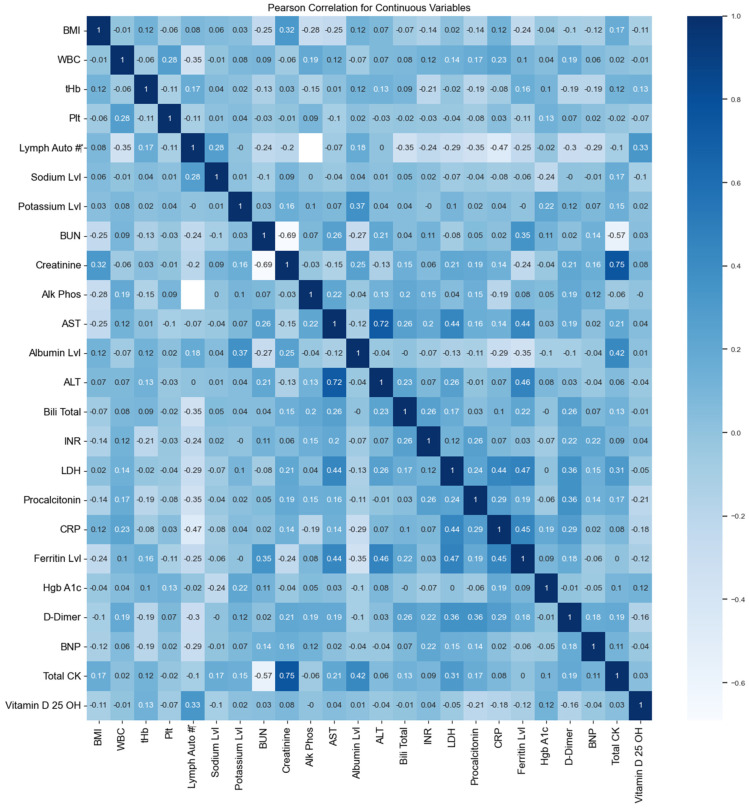
Correlation of the continuous features in the dataset.

The Pearson correlation coefficient was used to obtain the relationships between the continuous features. Cramér’s V was used to measure the association between the categorical features. Figure 5 shows the correlation of the continuous features; the heat map shows the correlation between twenty-four continuous features. A correlation value of 0.75 was recorded between both creatinine and total CK. Furthermore, a correlation of 0.72 was observed between total AST and ALT. Table 5 indicates that there is no association between age and nationality, while there is a weak association between age, nationality, and MEWS score.

### 3.3. Data Preprocessing

Efficient preprocessing of data can have a major effect on the reliability and quality of deep learning model results. It assists in guaranteeing that the data are accurate, in the right format, free of errors, and in line with the objectives of the modeling tasks [20]. 

To preserve data privacy, we anonymize the identity of the patients in the CXR images and the tabular data because it is not included in the analysis. In the following subsections, we discuss each preprocessing step for each item of the tabular data and the CXR images in detail and illustrate its main methods.

#### 3.3.1. Tabular Data 

The raw dataset contains tabular data that were obtained in their original, unprocessed form, and we cleaned and organized the data in a unified tabular structure in line with the Dubai COVID-19 data format.

Before addressing the missing values in the tabular data, the data type was checked to make sure that the data in the dataset were correctly formatted and that the data types were consistent. Since the presence of string values in the data is minimal, the appropriate action would be to convert these string values to null and then proceed as missing values.

As is well known, managing the missing values in medical data is not straightforward [21]. This is due to the nature and sensitivity of these data, where replacing the missing values with a value of 0 has different meanings in this field. For this reason, we predict the values for the features that have less than 75% of their values missing for each file. Due to the importance of all features and an inability to remove any of the existing ones, we combined all files and repeated the process for the features in which the percentage of missing values was higher than 75%.

Extreme Gradient Boosting (XGBoost) and Random Forest are machine learning models used to impute continuous and categorical missing values. For each class, the data are split into two parts: a training dataset (non-missing values) and a test dataset (missing values to be imputed). The XGBoost Regressor and Random Forest Regressor are used for continuous features, while categorical features are imputed using the XGBoost Classifier and Random Forest Classifier. Thereafter, box and bar plots are utilized to visualize the distribution of continuous and categorical values, respectively, and spot the outliers. The interquartile range (IQR) method is used to identify the outliers; values outside the range of the lower bound [q1 − 1.5 × iqr] and upper bound [q3 + 1.5 × iqr] are considered outliers. Considering the importance of each individual patient in the dataset, no patients were removed. The outlier values are replaced with “NaN” and then imputed using the XGBoost Regressor and Random Forest Regressor for continuous features. Subsequently, the features where the percentage of missing data exceeds 75% are returned and imputed; each column in this list is considered a target for imputation. The same approach is used to create an initial predictive model using the target column and common non-null features from the trainable datasets. Following each training cycle, a decision tree structure visualization of the model is created and saved. This visualization aids in understanding the model’s decision pathways. Once these predictions are made, the missing values in the original datasets are updated.

Then, applying a log transformation to the continuous columns in the dataset makes the data more normally distributed and standardizes the numerical values to scale them in the range of a mean of 0 and a standard deviation of 1, ensuring that the data are appropriate for subsequent modeling steps. Principal component analysis (PCA) is used to extract features from high-dimensional tabular data. Maximum likelihood estimation (MLE) is used to reduce the number of principle components from 644 to 218 components, significantly decreasing the complexity of the data while retaining the essential variance to focus on the most informative aspects of the data for modeling. The most important demographic and health-related data, in order of importance, are the following: age, BMI, MEWS score, nationality, and then gender. The top ten lab tests in order are INR, Total CK, D-Dimer, CRP, LDH, Albumin Lvl, BUN, Vitamin D 25 OH, and WBC test. The following medications were among the most important: Spironolactone, Granisetron, Colchicine, Sulfasalazine, Nifedipine, Gliclazide, Sodium Bicarbonate, Ferrous Sulfate, Metformin, and Pioglitazone, respectively.

#### 3.3.2. Images

All the CXR images were downloaded from picture archiving and communication systems (PACS). The radiology consultant was provided with files containing patient file numbers according to each class; the radiology consultant fetched all the CXR images of the specific range of years (e.g., COVID-19 from 2020 to 2022) that related to the listed patients. Each image was selected and labeled by the represented class based on matching the date when the patient visited the hospital and was diagnosed with the disease with the date that the CXR image was taken.

The CXR images were obtained in the DICOM (Digital Imaging and Communications in Medicine) extension. The MicroDicom DICOM viewer version (DM_PLATFORM_XRAY_GANAPATI_4.10.2_2020_FW41.1_158) was used to convert the images to the appropriate format of a JPG file [22].

To help the deep learning model focus on the chest area (especially the lungs), the images were manually cropped to guarantee that no tiny part of the lungs was removed in any way, cutting out a chest region and removing any other parts of the body that appeared in the image. Applying image enhancement is also important to improve the classification result. The ability of the gamma-correction-based technique to detect COVID-19 from CXR images outperforms other methods [23]. Trying different thresholds, the gamma threshold value (0.9) was chosen with the contrast enhancement threshold value (1.5) to enhance the contrast of the CXR images. Combining them enables a more thorough adjustment of the appearance and tonal range of the image. If *P* is the pixel value within the range [0,255], then *x* is the pixel’s grayscale value (*x* ∈ *P*). The output pixel vector of the gamma correction function *g*(*x*) is calculated with Equation (1).

(1) $$g\left(x\right)=255{\left(\frac{x}{255}\right)}^{1⁄\gamma (x)}$$

In addition, we apply image denoising using the total variation filter (TVF) method to remove the noise from the images. Based on the literature, combining contrast enhancement (gamma correction) and image denoising (TVF) approaches produces outstanding results on COVID-19 images [24]. Moreover, transformations are used to preprocess the images before the training phase. Due to dealing with CXR images from different resources and sizes, standardization was applied by resizing the images. The images were resized to 128 × 128 pixels, which gave a better result through the experiments. The images were converted to grayscale and the image pixel values were normalized to a range by dividing by 255 (the maximum pixel value for 8-bit images) and then converting to a tensor for integration with the TensorFlow framework.

#### 3.3.3. Eliminating Rib Shadows in CXR Images

A significant challenge in the study of chest radiographs is the invisibility of anomalies due to the superimposition of normal anatomical components, such as ribs, over the primary tissue under examination [25]. Therefore, it would be helpful to eliminate the ribs without losing any information about the original tissue when trying to increase the nodule visibility and identify nodules on a chest radiograph. For that reason, we tried to apply a method [26] for removing the rib shadows from the infected lungs to improve nodule detection and enhance the diagnostic process. A hybrid self-template approach was used wherein the algorithm tried to first identify the ribs. An unsupervised regression model was then used to suppress the identified ribs. We attempted to adapt the paper’s approach to our private dataset in order to achieve the same good results for rib elimination from their CXR images. 

After preprocessing the images, the lung area was defined using a Gaussian filter to extract the mask from each image. Rib detection was performed using a bilateral filter, and the result was then converted to grayscale using Extreme Level Eliminating Histogram Equalization (ELEHE) to improve the visibility of features in images. After that, Sobel edge detection was applied to the equalized image to define the edge thickness. Then, dilation was applied to merge nearby bright regions and increase their size; an opening operation was also performed on the dilated images. Erosion was applied to the opened images to shrink the bright regions and refine the image by reducing the size of the remaining bright regions after the opening operation. To prepare the images for parabola fitting, connected component analysis was performed on the images to define the connected components. The first (background) and last (foreground) components were excluded as they were not useful; then, fine tuning to the best number of connected components was performed. Thereafter, parabola fitting was calculated using Equation (2):

(2) $$f(x)=ax^{2}+bx+c$$

The most fitting connected components were considered, and all the curves were plotted on the images using polyline’s function. Finally, the rib region was acquired, shadow estimation was clearly defined, and suppression was performed by removing the shadows from an image, achieved by adjusting the pixel values in the shadow regions based on the average BGR color values. 

**Figure 6:**
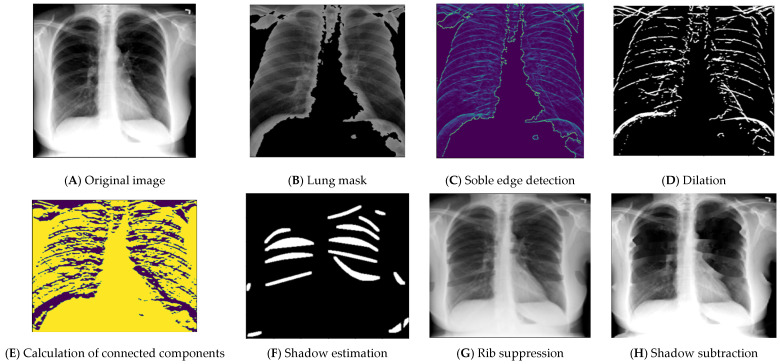
An example of the rib shadow elimination process and result.

Applying the previous approach to our private CXR images, as shown in Figure 6, delivers unacceptable results on most images. It removes a lot of nodules from the lung area, which affects the prediction results. It is important to note that the effectiveness of shadow suppression may depend on the characteristics of the images, which could be attributed to a lack of clarity of vision and sometimes the boundary of the lungs for most images, despite them being preprocessed. Testing on a variety of images is often necessary for a robust shadow removal model. For that reason, we decided not to apply rib elimination in this experiment.

### 3.4. Generating Synthetic Dataset

One potential pitfall to consider with this approach is the presence of imbalanced datasets. When there is a significant skew in the distribution of classes, with some classes having far fewer samples than others, the model can become biased towards the majority class. This means that the model might perform well in identifying the common class but struggle to accurately classify the less frequent ones. This bias can lead to misleading results and limit the generalizability of the approach to real-world scenarios with a more balanced class distribution. To address bias in model training, we need to balance the dataset to ensure that the number of instances for each class is roughly the same. Balanced datasets often lead to better model performance [27]. While the number of cases for classes in each level of the hierarchy structure are not balanced, we need to generate synthetic data to balance the dataset. Given that the most common augmentation methods used to increase the dataset do not fit with the type of dataset that is being used in this research, we choose GANs as the base model. A variety of versions of the model have been developed, each with a particular purpose [28].

A conditional tabular generative adversarial network (CTGAN) was used to generate synthetic tabular data. A CTGAN synthesizer is the generator that is responsible for creating synthetic data samples. It is initialized with the best epoch value for each data file that yields the optimal loss values, fits the synthesizer to the data, and generates synthetic data using the fitted synthesizer. The desired samples of records that we needed to generate were specified to achieve balance among each level of the dataset. The discriminator acts as a critic, aiming to distinguish between real data samples from the training set and the synthetic samples generated by the generator. The CTGAN generator consists of multiple fully connected layers with a leaky ReLU activation function and batch normalization to improve stability during training. The discriminator has the same structure, but it outputs a probability score between 0 and 1, indicating how likely the input data sample (real or synthetic) originates from the true data distribution. 

The evaluation of the quality of the synthetic data compared to the real data yielded superior results from synthetic data. The CTGAN-generated synthetic data demonstrated high fidelity, with overall quality scores of 96.21%, 97.26%, 98.46%, etc. For the respective datasets, this closely mimics the real data in terms of column shapes and pair trends. This high degree of similarity suggests that the synthetic data can accurately represent the real dataset, thereby minimizing the risk of introducing bias through unrepresentative samples. Furthermore, the successful evaluation metrics highlight that the synthetic data cover over 90% of the categories and continuous ranges found in the real data and adhere well to the minimum and maximum boundaries, ensuring that the integrity of data distributions and limits is maintained. The synthesis quality checks confirm that the generated data maintain a high level of quality and consistency with the original dataset, reducing the likelihood of introducing artificial trends or biases.

To generate fake CXR images from corresponding fake tabular data, we used a combination of tabular data and an image as inputs to a customized conditional generative adversarial network (CGAN). The model is trained by initially learning from the original tabular data and CXR images. Subsequently, it utilizes synthetic tabular data to generate corresponding synthetic CXR images. The generator is designed to produce 500 × 500 images, and the discriminator processes both tabular and image data. It is important to note that numerical-to-image synthesis with CGAN is a challenging task. The generator model has 10 layers, and the discriminator model has 8 layers; the count includes various types of layers, such as Dense, Conv2D, Conv2DTranspose, Batch Normalization, Dropout, ReLU, LeakyReLU, and Flatten layers. Both the generator and discriminator use the Adam optimizer with a learning rate of 0.0001, a beta_1 of 0.5, and a dropout rate of 0.2 used in the discriminator.

**Figure 7:**
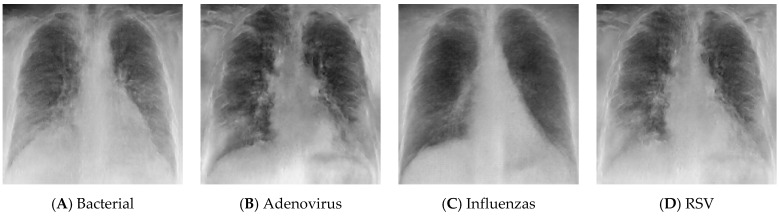
Samples of synthetic CXR images.

The model was trained for a number of epochs for each class to generate synthetic data. The model’s Structural Similarity Index Measure (SSIM) averaged around 0.67, which indicates a good degree of similarity to the original images. This value, although not perfect, suggests that the synthetic images capture much of the structural integrity and texture present in the real CXR images. Such a level of SSIM is often indicative of synthetic images. The higher peak signal-to-noise ratio (PSNR) was calculated to be higher than the average, which typically ranges between 20 and 40 dB for medical images. The PSNR value indicates that the synthetic images have a lower level of error or noise compared to the original images. The exact value of the PSNR that we targeted reflects a quality level that is generally accepted as good in the field of medical image analysis; some samples are shown in Figure 7. In the next section, we try to investigate how synthetic data improve classification accuracy, especially when a small amount of data is available.

## 4. Hierarchal Model Architecture

To develop a hierarchical classification by applying deep learning models, we adapted four pre-trained models to tackle the hierarchy classification process. It has been observed that Visual Geometry Group (VGG)-based and Residual Network (Resnet)-based models are widely utilized in this field and provide outstanding results [29,30]. We adopted VGG11 and Resnet18 as the basic models for this challenge because both are more suitable for the moderate size of our dataset. The dataset consisted of eight hierarchical paths that are shown in Table 6. The number of samples in the table represents only the number of cases in the original datasets. The details of all models are explained in the following subsections.

### 4.1. Hierarchical Convolutional Neural Network Based on the VGG Architecture

**Figure 8:**
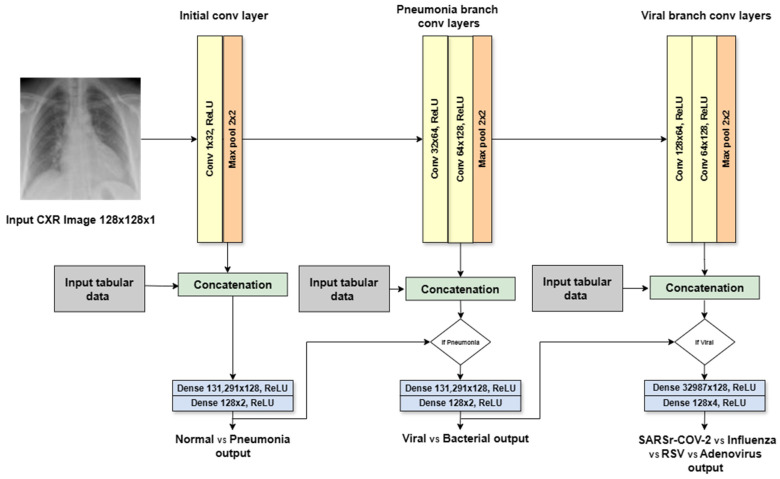
VGG-like multi-modal architecture.

A VGG-based neural network was mainly used as a deep learning multi-modal for the proposed method, with two architectures. The first architecture, called the VGG-like multi-modal, adapts the architectural principles of the VGG neural network architecture, which utilizes repetitive blocks of convolutional layers followed by Max Pooling layers to effectively extract features from CXR images. Our VGG-like multi-modal simplifies and tailors the original design for hierarchical decision making in pneumonia classification from CXR images, as shown in Figure 8. The adaptations were designed to process single-channel (grayscale) CXR images by modifying the first convolutional layer to accept a single input channel. The depth of the network was adjusted. The model includes fewer convolutional layers than some VGG models (e.g., VGG16, VGG19), making it effective for the targeted dataset. The model introduces branching points to make hierarchical decisions at different levels of pneumonia classification: normal vs. pneumonia, bacterial vs. viral pneumonia, and further subclassification of viral pneumonia. This hierarchical approach is novel and not present in the standard VGG architecture. After the initial shared convolutional layers, the network branches out to make specific decisions, with each branch having its own set of convolutional and fully connected layers tailored to its classification task. Considering the dataset size, the fully connected (ANN) layers in the branches are simplified compared to the Dense layers in the original VGG models, reducing the model’s complexity and the risk of overfitting on medical imaging datasets, which are typically smaller than ImageNet.

The input image size was changed to 128 × 128 pixels. The initial CNN layer for a first-level decision consists of one 2D convolution with thirty-two filters of size 1 × 1 and a kernel size of 3 × 3 using the ReLU activation, and Max Pooling with a kernel size of 2 × 2. The input for the initial layer is 1 channel, and the output is 32 channels. The flattened output from the initial CNN layer concatenated with the tabular data. These concatenated data are then passed to two hidden layers (Dense) of 128 neurons with ReLU activation. The final layer of the initial branch (decision #1) represents probabilities of normal/pneumonia classes. The pneumonia CNN layer for the second-level decision consists of two convolutional layers with the same kernel size, activation, and Max Pooling layer. The output (decision #2) represents probabilities of viral/bacterial classes. The viral branch for the third-level decision is similar to the pneumonia branch, but it has a different final layer with four output units (decision #3) corresponding to SARS-CoV-2, influenza, RSV, and adenovirus.

**Figure 9:**
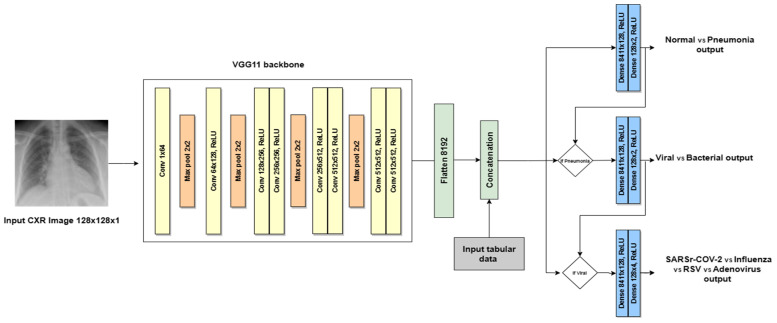
VGG-backbone multi-modal architecture.

The second architecture is the VGG-backbone multi-modal, which adapts the original VGG architecture by utilizing a pre-trained VGG11 model as a feature extractor, followed by three branches of fully connected layers (ANNs) for the hierarchical decision-making task. It has the same input image size: 128 × 128 pixels. The first convolutional layer was modified to handle a single input channel, applying 64 filters of size 3 × 3 followed by a Max Pooling layer with a kernel size of 2 × 2. This is followed by the second convolutional layer with 64 filters of size 3 × 3 followed by a Max Pooling layer with a kernel size of 2 × 2 using ReLU activation. This is followed by two convolutional layers and a Max Pooling layer with a kernel size of 2 × 2 using ReLU activation. This pattern of two convolutional layers followed by a Max Pooling layer is repeated for several blocks, progressively increasing the number of filters (typically doubling) to extract more intricate features. After the convolutional layers, the Flatten features are combined with the tabular feature and are passed to three separated branches of fully connected layers. These layers perform computations on all activations from the previous layers. The fully connected layers of the original architecture are replaced with custom layers designed to make hierarchical decisions specific to pneumonia classification, as shown in Figure 9. Each branch has a sequential block with two Dense layers and ReLU activation. The first Dense layer has 128 units. The second Dense layer has a specific number of units depending on the classification task. The first and second branches (decision #1 and decision #2) have two units (normal vs. pneumonia and viral vs. bacterial, respectively). The third branch (decision #3) has four units of the four viral subtypes (SARS-CoV-2 vs. influenza vs. RSV vs. adenovirus). This adaptation allows the models to focus on the most relevant features for each decision level. 

### 4.2. Hierarchical Convolutional Neural Network Based on the ResNet Architecture 

**Figure 10:**
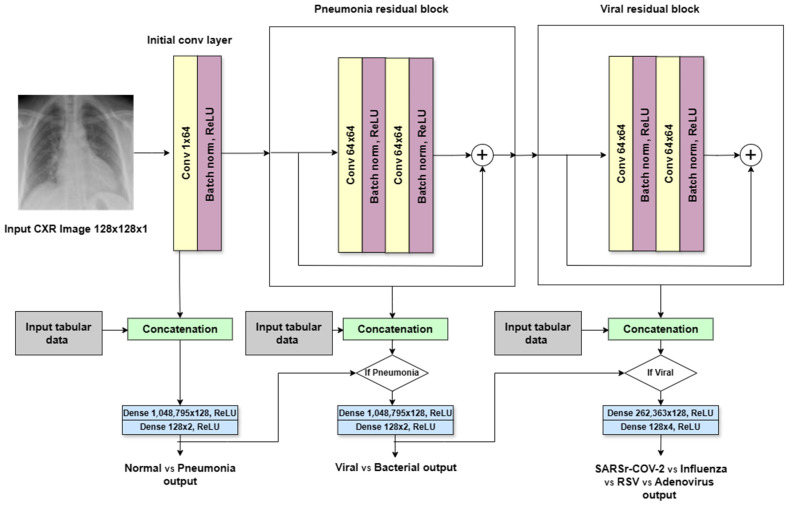
ResNet-like multi-modal architecture.

In addition to the VGG-based multi-modal, the ResNet-based multi-modal was also used as a deep learning multi-modal, with two architectures. The first architecture was the ResNet-like multi-modal, which was inspired by the ResNet architecture. The ResNet-like multi-modal adapts the ResNet architecture for the same hierarchical decision-making task in pneumonia classification, as shown in Figure 10. The input grayscale image size is 128 × 128 pixels. The key adaptations are modified in the first convolutional layer to accept grayscale images, reflecting the single-channel nature of CXR images. The model employs customized residual blocks that match the task’s complexity and data characteristics. Each block consists of convolutional layers with batch normalization and ReLU activation, similar to ResNet’s design, but the number and configuration of blocks are tailored to the pneumonia classification task. The initial block (decision #1) processes the entire CXR image to extract general features helpful for identifying patterns. It has one conventional layer that applies 32 3 × 3-sized filters with a stride of one and padding of one using ReLU activation, followed by the batch normalization layer. The second is the pneumonia block (decision #2), which aims to classify the CXR as viral or showing signs of bacterial infection. It consists of two conventional layers with 64 filters of size 3 × 3 followed by a batch normalization layer and ReLU activation. The last is the viral block (decision #3), which has the same specifications as the second block.

Similar to the VGG-like model, the ResNet-like model incorporates branching points for hierarchical classification decisions. This structure leverages the deep feature representation capability of ResNet while providing specialized decision paths for different classification levels. The introduction of skip connections in each block ensures effective training and feature propagation, even with the model’s depth. The model concludes with simplified, fully connected layers in each branch for decision-specific classification. Each branch of the network uses a sequence of two fully connected layers with a ReLU activation function in between. The first Dense layer in each branch has 128 units. However, the second Dense layer has a different number of units depending on the specific classification output it performs. Branch 1 (decision #1) focuses on the normal vs. pneumonia classification, which represents the two possible outcomes. The second branch (decision #2) assuming pneumonia is detected in the first branch; this branch classifies the pneumonia type as viral or bacterial. It utilizes a second Dense layer with two units, corresponding to the two classifications. The third branch (decision #3) aims to classify the specific viral subtype. It employs a second Dense layer with four units, representing the four possible viral subtypes (SARS-CoV-2, influenza, RSV, and adenovirus). Overall, the branching architecture leverages Dense layers with varying output sizes to handle different classification tasks within the overall pneumonia and viral subtype detection processes.

**Figure 11:**
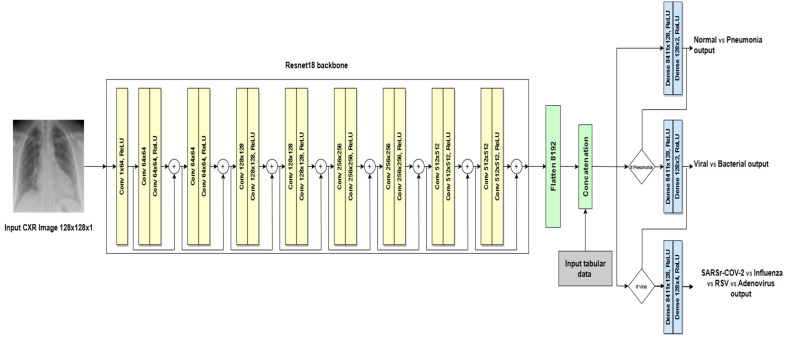
ResNet-backbone multi-modal architecture.

The ResNet-backbone multi-modal is the second architecture, which is an adaptation of the original ResNet architecture utilizing a pre-trained Resnet18 model as a feature extractor, followed by three branches of fully connected layers (ANNs) for the hierarchical decision-making task, as shown in Figure 11. It has a grayscale input image size of 128 × 128 pixels. The adaptations were designed to process single-channel (grayscale) CXR images by modifying the first convolutional layer to accept a single input channel. The model consists of 18 conventional layers with ReLU activation. The extracted features from the CXR images are flattened, transforming the 2D feature maps into 1D vectors suitable for fully connected layers. The flattened features from the CNN block combine image-derived features with additional tabular patient information. After utilizing the pre-trained network as a feature extractor, it employs three branches of fully connected layers (ANNs) for the hierarchical decision-making process. Instead of using standard fully connected layers, this architecture distributes the features into three branches, each containing a sequence of two Dense layers with a ReLU activation function in between. The first Dense layer in all branches has 128 units. The key difference lies in the second Dense layer, which adapts its number of units based on the classification task: two units for normal vs. pneumonia (decision #1), two units for viral vs. bacterial (decision #2, assuming pneumonia), and four units for the four viral subtypes (decision #3). This branching approach with custom layers allows the model to make hierarchical decisions tailored to each classification level. Adapting ResNet’s residual learning principle, the model efficiently learns features from CXR images, which is crucial for medical imaging tasks where interpretability and accuracy are paramount.

### 4.3. Training the Hierarchical Multi-Modal Methodology

We are now ready to train the four multi-modals using the hierarchical multi-modal approach. The pseudocode provided in Algorithm 1 illustrates the sequential training and classification strategy for a hierarchical model focused on pneumonia detection from CXR images and tabular data. This methodology enables the model to learn distinctive features relevant to each decision level, improving its ability to generalize and accurately classify new data.
**Algorithm 1.** Pneumonia Hierarchical Classification1:**Input:** dataset_path, num_epochs, batch_size2:**Output:** Trained Hierarchical Model3:1. Initialize transformations for dataset preprocessing4:2. Load and split dataset into training and testing sets5:3. Define model, loss function, and optimizer6:**Function** TrainModelForDecision(model, train_data, decision_point, loss_weights)7:  **For** each epoch in num_epochs do8:    **For** each batch in train_data do9:     Perform forward pass for the current decision_point10:     Compute loss using decision-specific loss_weights11:     Perform backward pass and update model parameters12:    
**End For**
13:  
**End For**
14:**End Function**15:Sequentially train model for each decision point in the hierarchy16:  a. **For** decision_point in [decision_1, decision_2, decision_3] do17:    i. Set appropriate loss_weights for the current decision_point18:    ii. Call TrainModelForDecision with the current decision_point19:    iii. Optionally adjust model for next decision_point20:  b. **End For**21:**Function** ClassifyImage(image, tabular data, model)22:  Perform model inference on the combined features 23:  Extract and return decision outcomes for each hierarchy level24:**End Function**25:4. Demonstrate classification with a sample image and tabular data using the trained model

The hierarchal multi-modal first determines whether the image shows signs of pneumonia. If pneumonia is detected, it then classifies the pneumonia as either viral or bacterial. If viral pneumonia is detected, the model further classifies the type of viral pneumonia. The hierarchical inference function returns a tuple of decisions, each corresponding to a level in the decision hierarchy. Algorithm 2 details the proposed inference with conditional flow in the form of pseudocode.
**Algorithm 2.** Inference with Conditional Flow1:**Input:** CXR images, Tabular data, trained model2:**Output:** Normal **OR** Pneumonia/Bacterial **OR** Pneumonia/Viral/(SARSr-CoV-2/Influenza/RSV/Adenovirus)3:**Function** Hierarchical_Inference (image, tabular data, model)4:decision_1_probs = model.forward_pass (image, tabular data, decision_point = ‘decision_1’) 5:decision_1 = ArgMax(decision_1_probs) # Normal vs. Pneumonia classification6:  **If** decision_1 is ‘Pneumonia’ **Then**7:    decision_2_probs = model.forward_pass (image, tabular data, decision_point = ‘decision_2’)8:    decision_2 = ArgMax(decision_2_probs) # Viral vs. Bacterial classification9:    **If** decision_2 is ‘Viral’ **Then**10:    decision_3_probs = model.forward_pass (image, tabular data, decision_point = ‘decision_3’)11:    decision_3 = ArgMax(decision_3_probs) # Subtypes of Viral Pneumonia classification12:    **Return** (‘Pneumonia’, ‘Viral’, decision_3) # SARSr-CoV-2/Influenza/RSV/Adenovirus13:     
**Else**
14:    **Return** (‘Pneumonia’, ‘Bacterial’, ‘N/A’) # No further subclassification15:    
**End If**
16:   
**Else**
17:    **Return** (‘Normal’, ‘N/A’, ‘N/A’) # No pneumonia detected, no further classification18:   
**End If**
19:**End Function**

The training methodology adopted for the VGG-like and ResNet-like multi-modals involves a sequential and focused approach, targeting one decision point at a time within the hierarchical structure of the problem. This approach ensures that the models learn to accurately classify at each level of decision making, from distinguishing between normal and pneumonia cases to identifying specific types of pneumonia, and so on. Focusing on the first decision point (normal vs. pneumonia), which distinguishes between normal and pneumonia cases, during this phase, the training process begins as the following:

- Train the VGG-like or ResNet-like model, focusing solely on the first decision point.
- Set the loss weight for the first decision point (e.g., normal vs. pneumonia) to 1.
- Set the loss weights for subsequent decision points (e.g., viral vs. bacterial, viral subtypes) to 0. This ensures that the model concentrates its learning on accurately classifying the initial coarse categories without being influenced by the more detailed classifications that follow.
- Train the model until it achieves satisfactory performance on the first decision to distinguish normal from pneumonia cases.
- The training proceeds to the next decision point (bacterial vs. viral). For this phase, the model’s weights from the previous training step are retained, ensuring continuity and leveraging learned features. The loss weight for the current decision is now set to a higher value (e.g., 0.9 for decision #2), while the loss weight for the first decision might be reduced (e.g., 0.1) to maintain its knowledge, and the loss weight for the third decision is set to 0. This process is repeated for each subsequent decision point, gradually shifting the model’s focus down the hierarchy.

Although the VGG-backbone and ResNet-backbone models are not hierarchical in architecture, the training process incorporates hierarchical principles to align with the structured decision-making process of the problem. For these models, the training methodology mimics the sequential focus used for the hierarchical models, adapting the learning process to emphasize one level of classification at a time. This structured approach ensures that the backbone models, which are powerful feature extractors due to their pre-trained weights, are finely tuned to the specific requirements of each decision point in the classification task.

While cases in hierarchical classification must follow a predefined hierarchy structure, local (top-down) and global (big-bang) are the two main approaches that can be used for addressing hierarchical classification [31]. Global and local approaches are also implemented in the hybrid approach, as in this work. From the local approach, the function proceeds in a top-down manner, starting with a broad classification (decision_1) and refining the classification based on subsequent decisions (decision_2 and decision_3). From a global perspective, the function considers the entire hierarchy of classifications as it defines the possible outcomes at each level and makes decisions based on the entire set of possibilities. Therefore, both local and global approaches are implemented in this hybrid approach, as explained.

Among the four architectures, ResNet-like has more computation time due to its complex structure. The VGG-backbone follows in terms of computational demands, while the ResNet-backbone and VGG-like models require less computation time.

### 4.4. Evaluation Metrics

To analyze the general classification performance, we must choose the right evaluation method. As mentioned, we addressed the class imbalance issue in the dataset by generating a significant amount of synthetic data. Since the data were now balanced, we decided to use macro-avg as the primary metric to calculate the mean evaluation metrics between the classes. The proposed models were assessed using common evaluation metrics such as accuracy, precision, sensitivity, and F1-score. The research targets hierarchal multiclass classification, using a 6 × 6-sized confusion matrix to output for values—true positive (TP), false positive (FP), false positive (FP), and false negative (FN)—used to calculate the following measures [32]:

Macro-average accuracy measures the average correctly predicted cases from each class to the total number of instances evaluated, as shown in Equation (3):

(3) Macro-average Accuracy= 1C×∑TPi +TNiTPi+TNi+FPi+FNi

Macro-average precision measures the average ratio of true positives among all predicted positives across all classes, as shown in Equation (4):

(4) Macro-average Precision= 1C×∑TPi TPi+FPi

Macro-average sensitivity measures the average ability of the model to correctly identify true positives across all classes, as shown in Equation (5):

(5) Macro-average sensitivity= 1C×∑TPi TPi+FNi

Macro-average F1-score combines both precision and recall into a single metric, providing an overall measure of the model’s performance in terms of correctly identifying true positives and minimizing false positives and negatives, as shown in Equation (6):

(6) Macro-average F1-score= 1C×∑2×TPi 2×TPi+FPi+FNi

where

*C* is the number of classes in the classification task;
TP*_i_* is the number of true positives for class *i*;
TN*_i_* is the number of true negatives for class *i*;
FP*_i_* is the number of false positives for class *i*;
FN*_i_* is the number of false negatives for class *i*.

## 5. Results and Discussion

The experiments were implemented using the Python 3.10.12 language and PyTorch 2.1.0+cu121 for the deep learning models. The training of the model was performed using a PC running the 64-bit Windows 11 Pro operating system. The PC had an Intel^®^ Core™ i7-10700 CPU @ 2.90GHz and 32GB of RAM (Intel, Santa Clara, CA, USA). Due to the limited size of some classes in the dataset (total of 4543 patients) and to obtain a more robust model, the data splitting approach was 70/30 for training and testing, respectively, using k-fold cross-validation with k set to five. Random searching within an iterative process was used to adjust the hyperparameter values of the models. This iterative approach drew inspiration from the value ranges employed in relevant research focused on similar problems and utilizing the same model architecture. Each model used the Adam optimizer with a learning rate of 0.001, a learning scheduler patience of three, an early stopping patience of five for the model without tabular data and fifteen for the model with tabular data, and cross-entropy loss as a loss function. The models were trained in ranges of 20 to 40 epochs with batch sizes of 32. The hierarchical multi-modal consistently performed well, given that the cross-validation score was consistent across all cross-folds.

**Figure 12:**
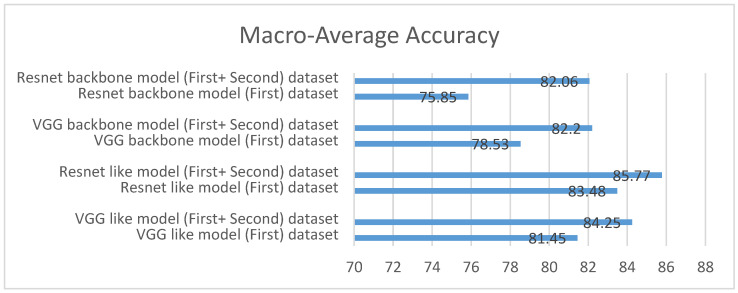
Comparison of macro-average accuracy for all models with and without a second dataset.

As mentioned in Section 4, four hierarchical deep learning multi-modals have been created to diagnose COVID-19 using CXR images and clinical tabular data and have been applied in many experiments. The first and second experiments were conducted to measure the performance of hierarchical deep learning models with and without a second dataset (used only CXR images), which is mentioned in Section 3, and before integrating the synthetic CXR images. Figure 12 shows the macro-average accuracy of the models before and after integrating the second dataset. The clear enhancement of all models’ performance after integrating the second dataset signifies a positive impact on the models’ ability to classify pneumonia accurately. The increased performance range (2.29–6.21%) indicates a significant improvement for all models. 

The third experiment was performed for all models after integrating the synthetic CXR images with the original CXR images from all datasets. Table 7 shows the results of all decisions at each level for each hierarchical classification schema. We observed that the best results for decision #1 and decision #2 (which are binary classification) were obtained using the Resnet-like model, while the best results for decision #3 (multi-classification) were obtained using the Resnet-backbone model. In addition, the results of the comparison of COVID-19 classification for each hierarchical classification schema are shown in Table 8. COVID-19 classification using the Resnet-backbone model is higher than other models, demonstrating an F1-score of 85.82% and an accuracy of 92.88%. Table 9 represents the macro-avg results that were achieved from all hierarchical classification models. Compared to the first and second experiments, the results improved very clearly for all models after integrating the synthetic CXR images to balance the dataset. The Resnet-like model achieved the best results among all models, with an F1-score of 92.65% and an accuracy of 92.61% for classifying all the classes.

In the last experiment, we applied the multi-modal approach by combining both the CXR images and tabular data. Compared to the third experiment, the results improved very clearly for all models after integrating the medical tabular data into the CXR images. This indicates the importance of adding medical data to CXR images for the diagnostic process and that depending only on the imaging modality does not achieve the required accuracy results. Key demographic factors that contribute to classifying pneumonia include the patient’s age, body mass, MEWS score (indicating overall clinical severity and vital signs), nationality, and gender. Additionally, in terms of lab tests, blood clotting, muscle damage, blood clots, inflammation, tissue damage, Albumin level, kidney function, vitamin D, and white blood cell count are the most crucial factors and help to determine whether pneumonia is present as well as to determine its type. Finally, medications for heart failure, nausea, arthritis, blood pressure, diabetes, acidosis, and iron deficiency are among the most important to consider. What was dispensed to the patient when they were infected with pneumonia indicates the most important symptoms of the side effects associated with the infection.

**Figure 13:**
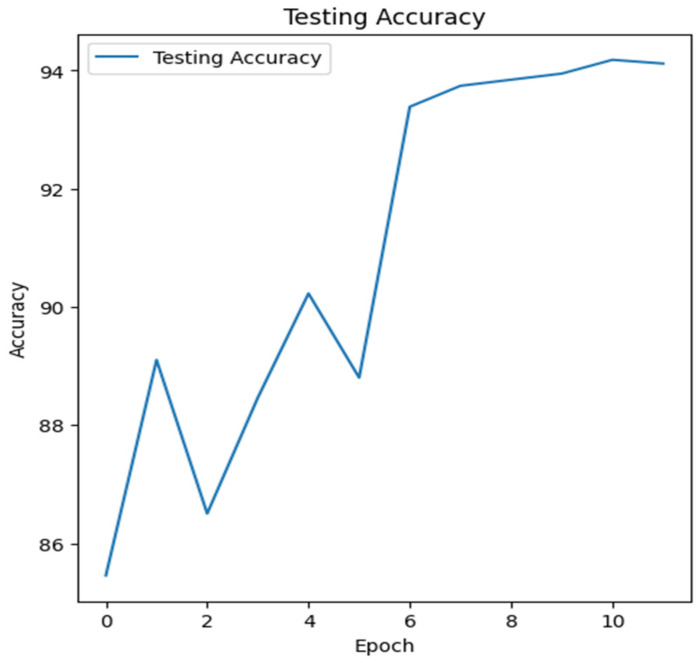
Testing accuracy (one-fold) against the number of epochs for the Resnet-backbone multi-modal in the last experiments.

Based on the training evaluation metrics, the Resnet-backbone hierarchal multi-modal performed the best among the four in this experiment. Figure 13 shows the testing (validation) accuracy/epochs for the Resnet-backbone multi-modal in one fold, which illustrates how the accuracy of the model improves over epochs. The VGG-based hierarchal multi-modal also shows good performance. Table 10 presents the results of different evaluation metrics of all decisions at each level for each hierarchical classification schema. We observed clear enhancement in all decisions. The best results for decision #1 were obtained using the Resnet-backbone multi-modal, while the best results for decision #2 were obtained using the VGG-backbone multi-modal. However, regarding decision #3, the results from the Resnet-based multi-modals were the best. The COVID-19 classification was also improved, as shown in Table 11, using the Resnet-based multi-modal. An accuracy of 93.72% and an F1-score of 88.24 was achieved using the Resnet-like multi-modal. Table 12 summarizes the macro-avg results that were achieved for this last experiment when integration between the CXR images and the tabular data produced superior diagnosis performance compared to the previous experiment. Resnet-backbone outperforms other multi-modals with an accuracy of 95.97% and an F1-score of 95.98%. 

**Figure 14:**
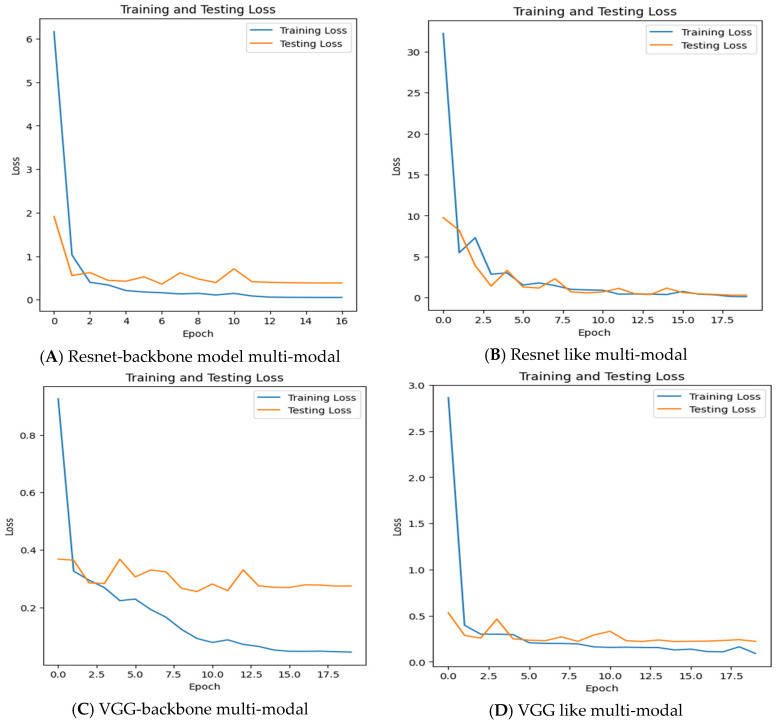
Training and testing loss against the number of epochs for each multi-modal in the last experiments.

Figure 14 shows the training and testing loss chart; the training loss starts high and steadily decreases over the epochs, which means that the models learn from the training data and improve their performance. The testing loss also decreases over the epochs, and it is close to the training loss. In this case, the two curves are very close, suggesting that there is no observed overfitting for any of the multi-modals.

**Figure 15:**
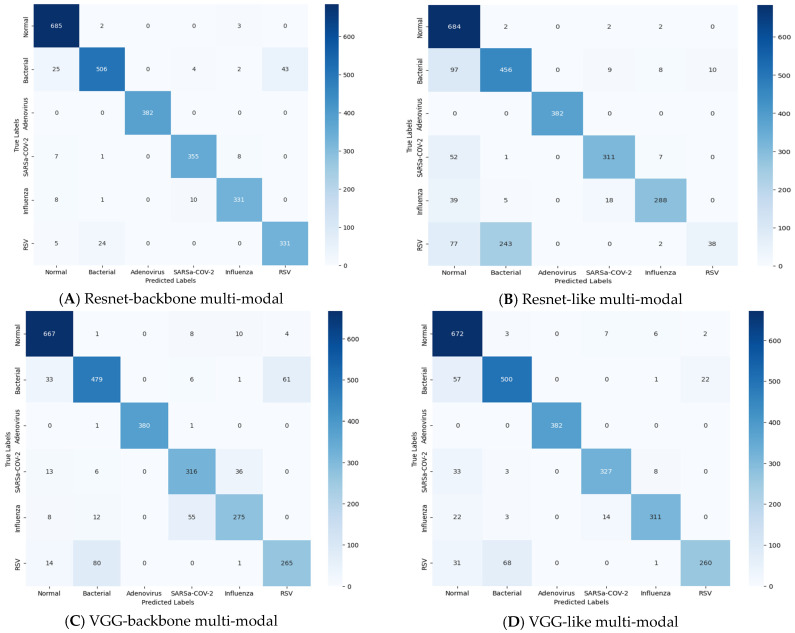
Confusion matrix for each multi-modal in the last experiments.

The 6 × 6 confusion-matrix plots for the four multi-modals are depicted in Figure 15. Instead of showing all eight possible classes, the confusion matrix only presents six. This is because some classes are grouped together. Imagine a hierarchy in which the classes (pneumonia and viral) are super classes of others (such as influenza). The confusion matrix focuses on the specific types (leaf nodes) because the overall pneumonia number is just the sum of its subtypes, and it presents the actual predicted cases. The horizontal axes correspond to the predicted classes, and the vertical axes correspond to the true classes, which represent the actual classifications. The diagonal cells in the confusion matrix represent the correct predictions (TP and TN). The off-diagonal cells represent incorrect predictions (FP and FN). From observing the number of false prediction cells, the Resnet-backbone model achieved a low overall misclassification rate of 4.03%. Bacterial pneumonia proved the most challenging with 74 falsely predicted cases, while the model perfectly classified all adenovirus cases. There were 5 falsely predicted cases for the normal class and 16 for COVID-19 classification, which is an acceptable outcome. There were 19 and 29 misclassification cases for influenza and RSV, respectively. Though viral pneumonia (including influenza, COVID-19, adenovirus, and likely RSV) saw 64 misclassifications, the overall pneumonia category naturally had a higher rate of 138 out of 1905 cases. Other models (VGG-backbone, Resnet-like, and VGG-like) exhibited slightly higher misclassification rates between 4.75% and 6.23%. All the multi-modals seem to perform well; however, the misclassification rate was very low, especially with the Resnet-backbone multi-modal.

**Figure 16:**
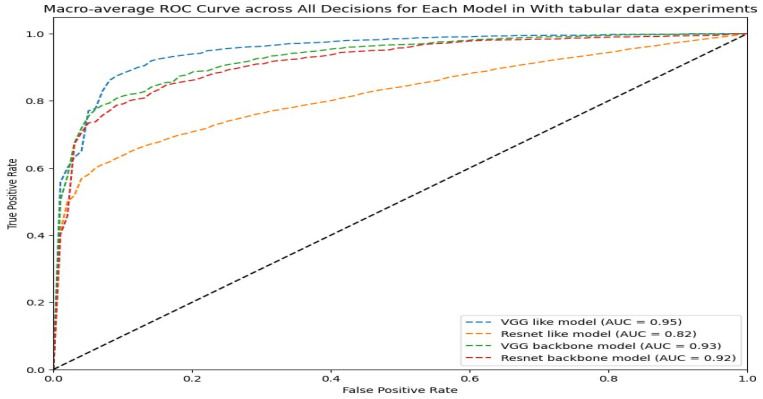
Macro-average ROC curve across all decisions for each multi-modal in the last experiments.

The macro-average ROC curve in Figure 16 demonstrates that the VGG-like multi-modal (AUC = 0.95) has the best overall performance across all classes, followed closely by the VGG-backbone and Resnet-backbone multi-modals (AUC = 0.93~0.92). Taking into consideration the other performance metrics, the Resnet-backbone multi-modal achieved superior performance in the classification process. 

While many studies have explored various approaches to the classification and identification of COVID-19, to our knowledge, no approach has attempted to classify COVID-19 by employing a hierarchical classification architecture that combines CXR image features with tabular medical data within a single model. This unique approach differentiates our work from existing research on COVID-19 classification, which is particularly evident when comparing the results of our proposed approach with those of similar works in the literature, as shown in Table 13. While binary classification and CT scan-based studies can achieve high accuracy, we opted not to compare our work to them for two reasons. Firstly, simplifying the problem into a binary classification might not reflect the complexities of real-world scenarios with more granular classifications. Secondly, CT scans, while valuable for diagnosis, can be impractical due to the limitations mentioned previously in the literature review. Our focus here is on more applicable, similar approaches.

Compared to previous research using a hierarchical structure for pneumonia classification [15], our proposed approach achieved better performance than the hierarchal model in this work. The overall performance of the model achieved a macro-average F1-score of 0.65, while the identification of COVID-19 cases specifically achieved an F1-score of 0.89 for this class. This study also faced limitations in the feature extraction phase. It relied on hand-crafted features, potentially missing more intricate patterns, and extracted features from a single modality. Additionally, the sample size was restricted, with only 1144 CXR images (1000 normal and a concerningly low 144 pneumonia cases, including COVID-19). This limited dataset might hinder the generalizability of the findings.

The multi-modal approach significantly outperforms previous work by Attaullah et al. [33] for classifying COVID-19 using a public dataset with five classes using the data of symptoms and CXR images, which achieved an accuracy of 78.88%. Also, the research in [34] combined clinical data with the CXR image features fed into a neural network architecture, achieving 73.2% accuracy and a 70.7% F1-score. While previous approaches have their merits, our findings yield demonstrably superior outcomes.

However, compared to flat classification studies that relied on CXR images only, our study surpasses previous work [35] that addressed a limited dataset (307 images) by employing a two-stage approach (data augmentation and deep learning). While their best result with GoogLeNet achieved 80.6% accuracy for multiclass pneumonia classification. In addition, the ensemble learning for the COVID-19 detection module in this research [36] categorized standardized CXR images into different classes, with the findings indicating that the developed deep learning system successfully identified COVID-19 pneumonia with an accuracy rate of 91.77% and an F1-score of 91.41%. Furthermore, it is noteworthy that our innovative approach surpasses these results, demonstrating superior performance in accurately predicting COVID-19 pneumonia through the integration of hierarchical classification architecture, combining CXR image features with tabular medical data within a unified model.

## 6. Conclusions

This paper proposes a novel approach for classifying COVID-19 and distinguishing it from other types of pneumonia and normal lungs using CXR images and medical tabular data in four different hierarchal architectures based on Resnet and VGG pre-trained models. This study used a private dataset obtained from King Khalid University Hospital and Rashid Hospital, containing a total of 4544 cases. This study aims to enhance the process of diagnosing COVID-19 and prove that combining CXR images with clinical data can achieve significant improvements in the hierarchal classification process. Overall, the performance metrics for all the hierarchal deep learning models were enhanced after combining the medical data with CXR images. Resnet-backbone achieved the highest performance with an accuracy of 95.97%, a precision of 96.01%, and an F-score of 95.98%. The proposed approach showed promising results, especially the hierarchal deep learning multi-modal. Our findings could aid in the development of better diagnostic tools for upcoming respiratory disease outbreaks. However, this study suffers from a data imbalance due to the lack of available patient medical data for some classes. This challenge affects the evaluation of the model’s performance. Generating a synthetic dataset makes the model more robust; however, it could also introduce biases or inaccuracies, potentially leading to unreliable results. To some extent, we are satisfied with the quality of our generated dataset so far, but we believe that there is room to enhance the quality of the synthetic dataset to optimize the model’s performance. In future work, we plan to explore more datasets from different resources, including different classes of pneumonia and lung diseases.

## Figures and Tables

**Table 1:** Patient characteristics for dataset (I).

Category	Value
Number of patients	3306
Gender	
Male	1584
Female	1722
Diagnosis	
SARSr-CoV-2	630
Normal	1272
Bacterial	248
Influenza	1120
RSV	21
Adenovirus	15
Age	
Range	0–103

**Table 2:** Patient characteristics for dataset (II).

Category	Value
Number of patients	1217
Gender	
Male	1070
Female	147
Diagnosis	
SARSr-CoV-2	1217
Age	
Range	19–87

**Table 3:** Description of attributes of the tabular data.

Feature Type	Feature Name	Description	Data Type
Demographic	Age	Patient’s age at the time of diagnosis.	Numerical
	Gender	Patient’s gender.	Categorical
	Nationality	Nation of origin of the patient.	Categorical
Vital signs	BMI	Patient’s body mass index.	Numerical
	MEWS Score	It is a calculation performed on a patient after checking their vital signs and AVPU score.	Categorical
Lab test	WBC	Test that measures the number of white blood cells.	Numerical
	tHb	Test that measures the total hemoglobin.	Numerical
	Plt	Test that measures the blood platelets.	Numerical
	Lymph Auto #	Test that measures the percentage of lymphocytes in your blood.	Numerical
	Sodium Lvl	Test of the sodium level in the blood.	Numerical
	Potassium Lvl	Test of the potassium level in the blood.	Numerical
	BUN	Test of the blood urea nitrogen.	Numerical
	Creatinine	Test that measures the level of creatinine in the blood.	Numerical
	Alk Phos	Tests that measure the level of alkaline phosphatase in the blood.	Numerical
	AST	Test that measures the levels of aspartate aminotransferase enzyme in the blood.	Numerical
	Albumin Lvl	Test that checks the amount of Albumin in the blood.	Numerical
	ALT	Test that measures the amount of alanine transaminase in the blood.	Numerical
	Bili Total	Test that measures the levels of bilirubin in your blood.	Numerical
	INR	The international normalized ratio is a blood test that measures how long it takes for blood to clot.	Numerical
	LDH	Test that measures the level of lactate dehydrogenase in the blood.	Numerical
	Procalcitonin	Test that measures the level of procalcitonin in the blood.	Numerical
	CRP	Test to check the C-reactive protein level in the blood.	Numerical
	Ferritin Lvl	Test that measures the amount of ferritin in the blood.	Numerical
	Hgb A1c	Test that measures the percentage of hemoglobin proteins in the blood that are coated with sugar.	Numerical
	D-Dimer	Test that measures D-dimer, which is a protein fragment that the body makes when a blood clot dissolves in the body.	Numerical
	BNP	A B-type natriuretic peptide test is for measuring the levels of a certain type of hormone in the blood.	Numerical
	Total CK	Test that measures the amount of creatine kinase in the blood.	Numerical
	Vitamin D 25 OH	Test that measures the level of active vitamin D in the blood.	Numerical
Medication	614 Medications	Total number of medications that were prescribed to the patient when visiting the hospital.	Categorical

**Table 4:** Patient’s medical information characteristics (*: data with statistical significance; a: chi-square test; b: Student’s *t*-test; c: Kruskal–Wallis H test).

Characteristics	COVID-19 (*n* = 1847)	Non-COVID-19 (*n* = 2677)	Overall (*n* = 4524)	*p* Value
Age (years)	48.9 ± 16.3	37.4 ± 20.95	42.65 ± 19.81	<0.001 *b
Gender				
Male	1402, 75.9%	1252, 46.8%	2655, 58.7%	<0.001 *a
Female	445, 24.1%	1424, 53.2%	1869, 41.3%	
Vital signs				
BMI	28.53 ± 10.86	39.03 ± 20.3	33.89 ± 17.19	<0.001 *b
MEWS score (normal)	581, 31.5%	2057, 76.8%	2638, 58.3%	<0.001 *c
MEWS score (low-risk)	603, 32.6%	365, 13.6%	968, 21.4%	
MEWS score (moderate-risk)	491, 26.6%	185, 6.9%	676, 14.9%	
MEWS score (high-risk)	106, 5.7%	59, 2.2%	165, 3.6%	
MEWS score (critical)	27, 1.5%	11, 0.4%	38, 0.8%	
Lab test				
WBC	9.69 ± 5.83	8.8 ± 4.46	8.98 ± 4.78	<0.001 *b
tHb	12.97 ± 2.18	13.03 ± 2.55	12.99 ± 2.32	0.729 b
Plt	232.81 ± 99.41	nan ± nan	232.81 ± 99.41	<0.001 *b
Lymph Auto #	1.3 ± 2.25	2.13 ± 1.44	1.92 ± 1.72	<0.001 *b
Sodium Lvl	135.77 ± 6.11	138.02 ± 3.65	136.99 ± 5.06	<0.001 *b
Potassium Lvl	4.14 ± 0.59	4.15 ± 0.49	4.15 ± 0.54	0.606 b
BUN	21.81 ± 20.96	5.13 ± 4.32	12.77 ± 16.75	<0.001 *b
Creatinine	36.81 ± 84.86	78.3 ± 104.39	59.27 ± 98.12	<0.001 *b
Alk Phos	99.38 ± 68.16	103.83 ± 79.9	102.72 ± 77.15	0.187 b
AST	71.5 ± 388.91	42.73 ± 222.26	54.68 ± 303.08	0.021 b
Albumin Lvl	27.04 ± 8.74	34.54 ± 5.2	32.68 ± 7.05	<0.001 *b
ALT	77.44 ± 114.05	44.16 ± 100.25	45.66 ± 101.99	<0.001 *b
Bili Total	11.18 ± 18.35	10.57 ± 19.74	10.72 ± 19.42	0.477 b
INR	1.08 ± 0.28	1.08 ± 0.26	1.08 ± 0.27	0.680 b
LDH	376.81 ± 331.01	363.38 ± 298.75	375.34 ± 327.59	0.553 b
Procalcitonin	1.67 ± 12.4	3.54 ± 12.36	1.91 ± 12.4	0.036 b
CRP	82.72 ± 84.94	68.91 ± 84.35	80.59 ± 84.97	0.009 b
Ferritin Lvl	1007.16 ± 2892.55	709.05 ± 6046.88	960.46 ± 3574.55	0.419 b
Hgb A1c	8.19 ± 5.34	7.47 ± 2.16	7.96 ± 4.58	0.001 b
D-Dimer	1.61 ± 2.51	2.37 ± 3.58	1.66 ± 2.61	0.030 b
BNP	2872.61 ± 14459.7	2505.09 ± 4094.67	2757.2 ± 12191.43	0.569 b
Total CK	126.63 ± 610.22	282.06 ± 505.17	170.01 ± 586.74	<0.001 *b
Vitamin D 25 OH	43.57 ± 33.94	42.62 ± 32.02	42.7 ± 32.16	0.818 b
Medication				
614 Medications (No)	1,231,221, 98.5%	1,623,034, 98.6%	3,269,812, 98.7%	1.000 a
614 Medications (Yes)	18,623, 1.5%	23,306, 1.4%	41,976, 1.3%	

**Table 5:** Correlation of the categorical features in the dataset.

Features	Correlation
(AGE, NATIONALITY)	0.000000
(AGE, MEWS Score)	0.196708
(NATIONALITY, AGE)	0.000000
(NATIONALITY, MEWS Score)	0.187111
(MEWS Score, AGE)	0.196708
(MEWS Score, NATIONALITY)	0.187111

**Table 6:** Dataset distribution for hierarchical classification.

Label Path	#Samples
Level#1	
Normal	1273
Pneumonia	3270
Level#2	
Pneumonia\Bacterial	248
Pneumonia\Viral	3165
Level#3	
Pneumonia\Viral\SARSr-CoV-2	1848
Pneumonia\Viral\Influenza	1281
Pneumonia\Viral\RSV	21
Pneumonia\Viral\Adenoviruses	15

**Table 7:** Results of decisions at each level for each hierarchical classification schema using only CXR images.

Models	Decision #	Accuracy	Sensitivity	Precision	F1-Score
VGG-like	Decision #1	94.92	94.92	95.12	94.98
	Decision #2	90.21	90.21	90.80	90.37
	Decision #3	88.95	88.95	89.06	88.98
Resnet-like	Decision #1	95.05	95.05	95.26	95.12
	Decision #2	92.82	92.82	92.90	92.85
	Decision #3	89.95	89.95	90.01	89.98
VGG-backbone	Decision #1	93.92	93.92	94.40	94.04
	Decision #2	88.41	88.41	89.51	88.67
	Decision #3	89.36	89.36	89.43	89.39
Resnet-backbone	Decision #1	93.90	93.90	94.52	94.04
	Decision #2	89.02	89.02	90.23	89.28
	Decision #3	90.32	90.32	90.49	90.33

**Table 8:** Comparison of COVID-19 classification results for each hierarchical classification schema using only CXR images.

Models	Accuracy	Sensitivity	Precision	F1-Score
VGG-like	90.82	80.80	83.57	82.17
Resnet-like	91.55	84.99	83.11	84.04
VGG-backbone	91.42	84.29	83.13	83.71
Resnet-backbone	92.88	82.37	89.56	85.82

**Table 9:** Comparison of macro-avg results for each hierarchical classification schema using only CXR images.

Models	Loss of Test Set	Accuracy	Sensitivity	Precision	F1-Score
VGG-like	0.43	91.36	91.36	91.66	91.45
Resnet-like	0.37	92.61	92.61	92.72	92.65
VGG-backbone	0.37	90.56	90.56	91.11	90.70
Resnet-backbone	0.30	91.08	91.07	91.75	91.22

**Table 10:** Results of decisions at each level for each hierarchical classification schema using CXR images and tabular data.

Models	Decision #	Accuracy	Sensitivity	Precision	F1-Score
VGG-like	Decision #1	93.63	93.63	94.67	93.83
	Decision #2	96.06	96.06	96.15	96.09
	Decision #3	91.62	91.62	91.70	91.65
Resnet-like	Decision #1	95.90	95.90	96.25	95.98
	Decision #2	93.29	93.29	93.89	93.41
	Decision #3	93.38	93.38	93.44	93.41
VGG-backbone	Decision #1	97.66	97.66	97.68	97.67
	Decision #2	96.68	96.68	96.80	96.71
	Decision #3	91.40	91.40	91.49	91.45
Resnet-backbone	Decision #1	98.13	98.13	98.17	98.15
	Decision #2	96.42	96.42	96.49	96.44
	Decision #3	93.35	93.35	93.38	93.36

**Table 11:** Comparison of COVID-19 classification results for each hierarchical classification schema using CXR images and tabular data.

Models	Accuracy	Sensitivity	Precision	F1-Score
VGG-like	92.30	85.71	82.64	84.15
Resnet-like	93.72	88.69	87.79	88.24
VGG-backbone	91.88	83.51	84.62	84.06
Resnet-backbone	93.89	87.96	86.98	87.47

**Table 12:** Comparison of macro-avg results for each hierarchical classification schema using CXR images and tabular data.

Models	Loss of Test Set	Accuracy	Sensitivity	Precision	F1-Score
VGG-like	0.23	93.77	93.77	94.18	93.86
Resnet-like	0.20	94.19	94.19	94.53	94.26
VGG-backbone	0.24	95.25	95.25	95.32	95.27
Resnet-backbone	0.33	95.97	95.97	96.01	95.98

**Table 13:** Comparison to related studies applying similar approaches.

Model	Methodology		Accuracy	F1-Score
	Hierarchal	Multi-Modal		
Pereira et al. [15]	Yes	No	-	65%
Attaullah et al. [33]	No	Yes	77.88%	-
Cheng et al. [34]	No	Yes	73.2%	70.7%
Loey et al. [35]	No	No	80.56%	82.32%
Rajaraman et al. [36]	No	No	91.77%	91.41%
Proposed Model	Yes	Yes	95.97%	95.98%

## Data Availability

The data presented in this study are available on request from the corresponding author due to (ethical reasons).
